# I know that I know. But do I know that I do not know?

**DOI:** 10.3389/fpsyg.2023.1128200

**Published:** 2023-02-23

**Authors:** Leona Polyanskaya

**Affiliations:** ^1^Georg-August-Universität Göttingen, Allgemeine Sprachwissenschaft, Göttingen, Germany; ^2^CIBIT-Coimbra Institute for Biomedical Imaging and Translational Research, Coimbra, Portugal; ^3^Faculty of Psychology and Educational Sciences, University of Coimbra, Coimbra, Portugal

**Keywords:** metacognition, decision confidence, retrospective confidence judgments, error detection, conscious awareness, metacognitive bias

## Abstract

Metacognition–the ability of individuals to monitor one’s own cognitive performance and decisions–is often studied empirically based on the retrospective confidence ratings. In experimental research, participants are asked to report how sure they are in their response, or to report how well their performance in high-level cognitive or low-level perceptual tasks is. These retrospective confidence ratings are used as a measure of monitoring effectiveness: larger difference in confidence ratings assigned to correct and incorrect responses reflects better ability to estimate the likelihood of making an error by an experiment participant, or better metacognitive monitoring ability. We discuss this underlying assumption and provide some methodological consideration that might interfere with interpretation of results, depending on what is being asked to evaluate, how the confidence response is elicited, and the overall proportion of different trial types within one experimental session. We conclude that mixing trials on which decision confidence is assigned when positive evidence needs to be evaluated and the trials on which absence of positive evidence needs to be evaluated should be avoided. These considerations might be important when designing experimental work to explore metacognitive efficiency using retrospective confidence ratings.

## Introduction

Living beings, including humans, constantly monitor environment and their own cognitive states in order to evaluate their past decisions (Kepecs et al., 2008; Smith, 2009). This is known as metacognitive monitoring – ability to evaluate one’s own cognition – and it is based on error-detection mechanisms. Meta-monitoring is an important component of metacognition because it lays the foundation for meta-control, or adjusting future behavior in accordance with goodness of past decisions and the ratio of resolved/retained uncertainty about the state of the world and mind (Nelson and Narens, 1990; Drigas and Mitsea, 2020). Meta-monitoring relies on estimating the probability of an error on each decision (Ordin et al., 2020). If the estimated probability of an error is high, then people tend to assign lower confidence to their decisions than in the cases when estimated error probability is low. Hence, confidence ratings assigned tend to better discriminate between correct and incorrect decisions of those individuals who are better at estimating the probability of committing an error, i.e., better metacognitive monitoring skills. Efficient metacognition is reflected in larger difference in average confidence ratings assigned to correct and incorrect responses.

Metacognition is not necessarily correlated with cognitive performance (Flavell, 1979; Nelson, 1996), leading to under- or overconfidence bias. Some individuals may be very good at a particular cognitive task without realizing that their performance is high and thus assigning low confidence to their answers. Other individuals, on the contrary, may perform in the same task poorly without realizing it. Regardless of how well these individuals perform in cognitive tasks, their metacognitive skills are poor. On the other hand, individuals with good metacognitive abilities do not have to perform a cognitive task at a high level. Good metacognition is reflected in being able to realize how well the task is performed and adjust confidence ratings accordingly (Smith et al., 2003; Persaud et al., 2007). Metacognitive efficiency is studied most frequently by explicitly asking people in experimental setting to report how sure they are in their response on each trial. These confidence ratings are then used to measure metacognition. Three important concepts need to be distinguished in the study of metacognition: metacognitive sensitivity, metacognitive efficiency, and metacognitive bias, which will be defined below.

Metacognitive sensitivity is the accuracy with which participants discriminate between potentially correct and incorrect decisions. The percentage of correct responses on trials to which higher confidence is assigned, tends to be higher than the percentage of correct decisions to which lower confidence is assigned. The percentage of correct decisions on trials assigned the lowest confidence ratings should be at the chance level, given that overall performance (average accuracy of decisions) is above chance.

Metacognitive efficiency reflects how well confidence ratings discriminate between correct and incorrect responses. Efficient metacognition manifests as bigger differences in confidence between correct and incorrect decisions. In laboratory settings, this is often limited by the confidence rating scale. If participants are asked to report whether they are sure or not sure about a given answer using a binary scale, estimating metacognitive efficiency as the difference in confidence assigned to correct and incorrect responses becomes methodologically more challenging.

Metacognitive bias is the general tendency of an individual to assign higher or lower confidence ratings to his decisions. Metacognitive bias can expand or contract the scale for shifting confidence ratings up or down to reflect fluctuations in the degree of decision confidence. In extreme cases, over- or under-confidence can limit the discriminative aspect of confidence: when an under-confident individual correctly estimates that the likelihood of an error in a particular case is high, he may not be able to assign a lower confidence rating to another response because the base reference for his confidence is already at the lowest level. The opposite logic might also be true for over-confident individuals, who tend to assign ratings at ceiling, and are not able to push the ratings higher on trials where they estimate the likelihood of an error to be very low.

Since task performance and metacognitive bias can influence metacognitive sensitivity and efficiency (Galvin et al., 2003; Fleming and Lau, 2014; Rouault et al., 2018), Maniscalco and Lau (2012) proposed using a signal detection analytic approach. The basic idea behind this approach is that cognitive hits and correct rejections, to which high confidence rating is attached, are considered to be metacognitive hits, and cognitive hits and correct rejections, to which low confidence is attached, are considered metacognitive misses. Cognitive false alarms and misses with high confidence are metacognitive false alarms, and cognitive false alarms and misses with low confidence are metacognitive correct rejections. Confidence ratings do not have to be binary, leading to more precise modeling, as described below.

Metacognitive sensitivity is estimated as task performance (D’) that would lead to the observed ROC curve for confidence ratings, given the absence of imprecision in assigned confidence ratings (modeling an ideal observer for confidence estimates). This fitted D’ is referred to as meta-D’ and may be higher or lower than D’, correspondingly signaling better or worse metacognitive sensitivity. If metacognitive judgments and cognitive decisions are based on partially parallel processing streams (Fleming and Daw, 2017), participants can perform at chance in a cognitive or perceptual task, yet exhibit high metacognitive sensitivity, meaning that their confidence ratings will discriminate correct and incorrect decisions. Metacognitive efficiency within this framework is defined as metacognitive sensitivity relative to individual task performance (e.g., M-ratio, measured as meta-D’/D’ or M-difference, measured as meta-D’-D’). Meta-D’ shows how accurately correct and incorrect decisions are discriminated, while M-ratio shows how well confidence tracks performance on a particular task given an individual level of performance on this task. This then allows comparing meta-efficiency across tasks of different difficulty, in different domains and modalities (important is that the task structure remains the same across modalities and domains, Ruby et al., 2017).

While this approach has clear advantages (e.g., Maniscalco and Lau, 2012; Fleming, 2017), it is important to also be aware of its limitations. Metacognitive hits include task cognitive hits and correct rejections with high confidence, placing, for example, equal weight on cognitive correct responses, regardless of whether they are given based on positive evidence (detection of signals, i.e., hits) or absence of evidence (signals not present and not detected, i.e., correct rejections). However, Meuwese et al. (2014) showed that metacognition is superior on trials that require estimating positive evidence compared to trials that require estimating absence of evidence. Kanai et al. (2010) showed that in some cases cognitive misses and correct rejections are not discriminated by confidence ratings, while hits and false alarms are discriminated. That said, the structure of the task (Ruby et al., 2017) and individual decision making strategies (explore vs. exploit; reject vs. accept, Kanai et al., 2010; Meuwese et al., 2014) might lead to multiple individual differences in metacognitive sensitivity and efficiency, as measured by meta-D’ and M-ratio.

In this report, we will look at discriminability of confidence ratings between correct and incorrect trials given based on presence and absence of evidence in an artificial language learning task with a yes/no recognition test. The task involves familiarizing people with a continuous sensory input with embedded recurrent discrete constituents. People detect and memorize these constituents during familiarization, and then they are subject to a recognition test, when they hear or see a token and need to respond whether this token is a constituent from the familiarization input or not. To measure metacognition, people are asked to assign confidence rating upon responding “yes” or “no” on each trial. This is a tricky test because on presenting the actual tokens from the familiarization input, participants need to estimate how sure they are in what they know. By contrast, on trials when foils are presented, participants’ confidence ratings reflect how sure they are in what they do not know. In SDT approach, however, both types of responses are used within one framework. But we can calculate individual difference between correct and wrong responses separately for foils and actual constituents, hence tapping on whether people “know what they do not know” (on trials with foils), and whether metacognitive processing on trials with foils and actual tokens differs. This might have important methodological considerations for future experimental designs.

## Method

The material for analysis was the same as described in details in Ordin et al. (2021). No experimental data was collected specifically for this study, an existing dataset (completely anonymized) was used, the ethical approval was obtained prior to collecting the primary dataset for the original study. For the readers’ convenience, the material and the procedure is outline below, without details, which are presented in the original article. I used the data collected on 48 Spanish-Basque bilinguals from students’ population at the university of the Basque country in Donostia-San Sebastian, Spain.

The data was obtained by running an artificial language learning experiments to investigate efficiency of statistical learning in the visual and auditory modalities on linguistic and non-linguistic material (semi-linguistic stimuli in the original dataset were not used in this analysis). The study was designed so that each participant performed all experiments, in a counter-balanced order.

For linguistic material, recurrent triples of syllables (further referred to as words) were embedded into a syllabic stream and presented *via* headphones in the auditory modality. In the visual modality, a different set of syllables was used to make another set of tri-syllabic words. Syllables were presented one by one in the middle of the screen. People listened/watched the familiarization sequence, and their task was to detect and memorize the words of this artificial language (explicit instructions were given as to what they will be tested on following the familiarization phase). Upon familiarization, we played *via* headphones or presented visually a tri-syllabic sequence. Participants had to report whether the sequence is a word from the artificial language or not, and how sure they were in their response (confidence rating was collected on a 4-point scale).

For non-linguistic material, we used fractals in the visual modality and environmental sounds in the auditory modality. The sounds/fractals were arranged into recurrent triplets embedded into familiarization input, and participants were explicitly instructed to detect and memorize these sequences. A yes/no recognition test followed.

For the recognition test in the auditory modality, eight words/sequences were created. The tokens for the test represented either recurrent sequences from the familiarization input (aka words) or foils. On foils, the transitional probabilities between separate elements (syllables/fractals/sounds) were 0% (i.e., the consecutive elements in the foils never occurred consecutively in the familiarization input). Eight foils preserved the ordinal position of elements, and eight foils violated the ordinal position of the elements in the words/sequences (i.e., if a particular element was used in the unit-initial position, it could only be used in the foil-medial or foil-final position).

In the visual modality, the number of words and foils was reduced by two. The order of sessions (modalities*domains) were counterbalanced across participants. During the tests, each token was used twice, yielding 48 trials in the auditory modality and 24 trials in the visual modality on each type of material.

## Results

Data from one participant was discarded because he always gave the same confidence rating across all trials. The remaining data was screened for outliers (defined as data values exceeding 3SE deviations from the mean in z-transformed scores) and for deviations from normality (using Kolmogorov–Smirnov tests). Neither significant deviations from normality nor extreme outliers capable of distorting the test results were detected.

### Analysis of metacognitive sensitivity

To analyze metacognitive sensitivity, we calculated the percentage of correct trials for all trials on which participants assigned high vs. low confidence ratings (as a number of participants did not use extreme confidence ratings at all, we lumped together all responses with confidence ratings 3-“sure” and 4-“absolutely sure” as high-confidence trails, and responses with confidence ratings 2-“not very sure” and 1-“unsure” as low-confidence trails). Here, we calculated the number of responses to which high or low confidence was assigned, and the number of correct responses among these responses, and calculated the ratio multiplied by 100. If a participant gave only 5 responses with high confidence, but all 5 responses were correct (100%), his metacognitive sensitivity was considered to be higher than that of a participant who gave 20 responses with high confidence, but only 10 were correct (50%).

**FIGURE 1:**
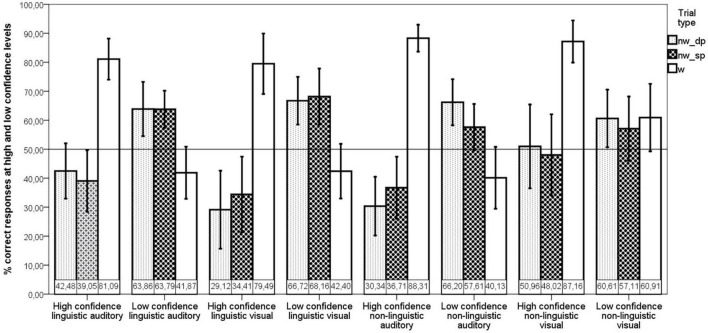
Meta-sensitivity measured as the percentage of correct responses for high and low confidence levels. If a participant gave only 5 responses with high confidence but all 5 responses were correct (100%), his metacognitive sensitivity was considered to be higher than that of a participant who gave 20 responses with high confidence, with only 10 correct (50%). Meta-sensitivity was calculated separately in the visual and auditory modalities, linguistic and non-linguistic domains, and on three different token types: random foils (nw_dp), ordered foils (nw_sp) and triplets (w). The horizontal line represents performance at the chance level.

The difference in the percentage of correct responses with high vs. low confidence ratings was significant for all token types (both linguistic and non-linguistic, both in visual and auditory modalities). On triplets, as predicted, responses to which high confidence is assigned are more likely to be correct than responses to which low confidence is assigned. On foils, although, the trend is the opposite: responses with low confidence are more likely to be correct than those with high confidence. This pattern is evident in Figure 1. All paired 2-tailed *t*-tests comparing the number of correct responses per confidence level in each modality and domain were significant (*p* < 0.0005 after Bonferroni correction), except *t*-tests for both types of non-linguistic foils in the visual modality (*p* > 0.5 before correcting for multiple comparison), also confirming that people are not sensitive to the likelihood of an error when they need to estimate how likely it is that they do not know something (evaluate absence of knowledge).

A more insightful result section below is related to metacognitive efficiency.

### Analysis of metacognitive efficiency

**FIGURE 2:**
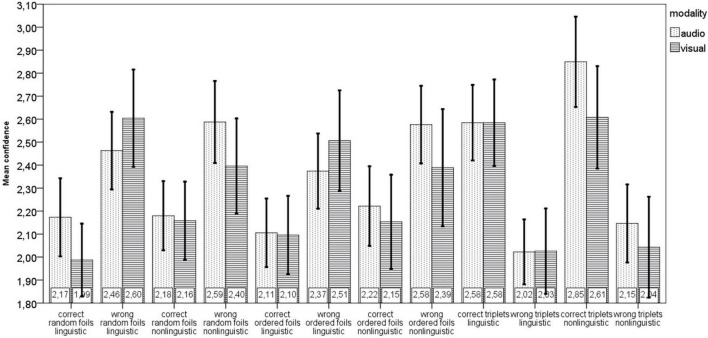
Confidence ratings assigned to correct and incorrect responses for different token types (random and ordered foils and triplets), stimulus types (linguistic and non-linguistic), and modalities (visual and auditory).

People who exhibit equally high metacognitive sensitivity may nevertheless differ in metacognitive efficiency, i.e., in the magnitude of the difference in confidence ratings assigned to correct and incorrect responses. Figure 2 shows that correct responses are assigned higher confidence than incorrect responses only on trials with triplets, while on trials with foils, higher confidence is more often assigned to incorrect than correct responses. A series of two-tailed *t*-tests showed that the differences in mean confidence assigned to correct and incorrect responses were significant for all token types in both modalities and for both stimulus types – linguistic and non-linguistic - with all *p*-values, corrected, <0.0005. The only exceptions where this difference was not observed were for foils in the non-linguistic domain in the visual modality (corrected, *p* = 0.72 for ordered foils and *p* = 0.12 for random foils). As metacognition is evidenced by assigning a higher confidence rating to correct than to incorrect responses (Galvin et al., 2003; Persaud et al., 2007; Maniscalco and Lau, 2012), the data suggests that metacognitive processes did not operate on the trials in which foils were presented. This conclusion agrees with the analysis that revealed metacognitive sensitivity only on trials in which triplets were presented.

Overall, the data is in line with Kanai et al. (2010) and Meuwese et al. (2014), showing that metacognitive sensitivity is higher when people need to estimate how confident they are in what they know. Our results are even stronger suggesting that metacognition fails when people need to estimate their confidence in absence of evidence.

**FIGURE 3:**
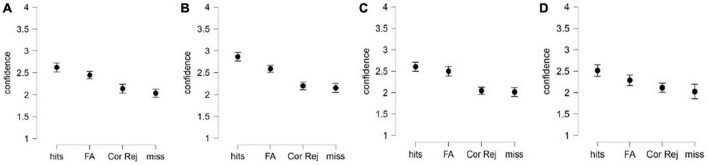
Confidence ratings assigned to correctly endorsed constituents (hits, “yes” responses), incorrectly endorsed foils (false alarms, “yes” responses), correctly rejected foils (correct rejections, “no” responses), and incorrectly rejected constituents (misses, “no” responses). Confidence ratings are represented separately for auditory **(A)** linguistic material, **(B)** non-linguistic material and visual, **(C)** linguistic material, and **(D)** non-linguistic material modalities. Error bars stand for 95%CI.

Earlier studies showed that on trials, in which the test tokens were endorsed, participants tend to assign higher confidence than on trials, in which the test tokens were rejected (Kanai et al., 2010; Maniscalco and Lau, 2014; Meuwese et al., 2014). To verify whether this pattern is observed in our sample, the data was re-analyzed conditional on the response type (*yes* vs. *no*), with *response type* and *correctness* as within-subject factors. In audio modality, the analysis on linguistic material revealed a significant effect of *correctness*, *F*(1,44) = 15.06, *p* < 0.001, η^2^_*p*_ = 0.255; and of *response type F*(1,44) = 49.01, *p* < 0.001, η^2^_*p*_ = 0.527, with insignificant interaction between the factors, *F*(1,44) = 0.92, *p* = 0.34, η^2^_*p*_ = 0.02. For each response type, correct responses were assigned higher confidence than wrong responses (confidence on *hits* was higher than on *false alarms*, and confidence on *correct rejections* was higher than on *misses*). On non-linguistic material in audio modality, the pattern was the same: a significant effect of *correctness*, *F*(1,46) = 14.299, *p* < 0.001, η^2^_*p*_ = 0.355; and of *response type F*(1,46) = 67.64, *p* < 0.001, η^2^_*p*_ = 0.59; yet the interaction between the factors was also significant, *F*(1,46) = 15.18, *p* < 0.001, η^2^_*p*_ = 0.25. The interaction is revealed in significant difference in confidence ratings between hits and false alarms, and lack of significant difference in confidence ratings between misses and correct rejections. These patterns are displayed on Figures 3A, B, for linguistic and non-linguistic material correspondingly.

In the visual modality on linguistic material, the analysis showed a significant effect of *response type F*(1,43) = 62.99, *p* < 0.001, η^2^_*p*_ = 0.59, while neither effect of *correctness*, *F*(1,43) = 2.41, *p* = 0.128, η^2^_*p*_ = 0.05, nor interaction between the factors, *correctness*, *F*(1,43) = 1.04, *p* = 0.314, η^2^_*p*_ = 0.02 turned out significant. On non-linguistic material in the visual modality, the pattern is identical to what we observed in the visual modality, with significant effect of *correctness*, *F*(1,43) = 9.38, *p* = 0.004, η^2^_*p*_ = 0.202; and of *response type F*(1,43) = 13.34, *p* < 0.001, η^2^_*p*_ = 0.265, and with insignificant interaction between the factors, *F*(1,43) = 1.82, *p* = 0.185, η^2^_*p*_ = 0.05. These patterns are displayed on Figures 3C, D, for linguistic and non-linguistic material correspondingly.

Higher confidence on correct responses than on incorrect responses on the constituents extracted from the familiarization sensory input and the reverse pattern on foils is possible if the participants exhibit a lenient response criterion on the cognitive task (i.e., if the tendency to endorse the presented test token is stronger than the tendency to reject the tokens, irrespective of their correctness). Given that each test token is presented twice during the recognition test, participants might develop a lenient criterion *via* familiarization with the test tokens after the first presentation. As “yes” responses tend to attract higher confidence compared to “no” responses, the lenient criterion may lead to higher confidence on *hits* (endorsed constituents from the sensory input) than on *misses* (rejected constituents from the sensory input) and lower confidence on *correct rejections* (rejected foils) than *false alarms* (endorsed foils). To consider this possibility, we calculated the response bias using the classical SDT approach. Positive bias signals an overall tendency to endorse items and negative bias signals an overall tendency to reject items. A score of 0 indicates no bias, hence significant deviations from 0 (using four one-sample *t*-tests, separate for each material type and perceptual modality) reveal the overall tendency to accept or reject the test tokens (the normality assumption was tested by the Shapiro–Wilk test).

**FIGURE 4:**
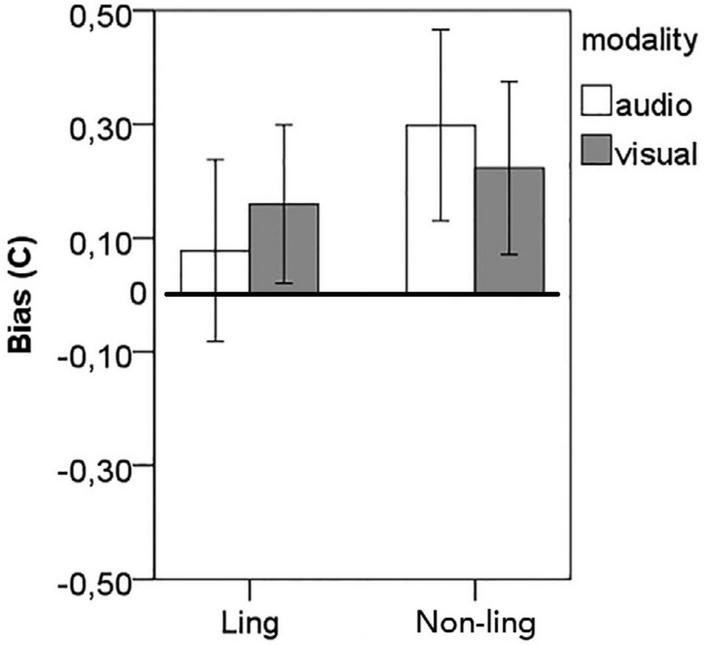
Response bias. A score of 0 indicates no bias, positive bias reflects a tendency to accept (endorse) test tokens (a tendency to respond “yes”). Error bars stand for 95%CI.

For linguistic material, the difference from zero was not significant, *t*(47) = 0.98, *p* = 0.331, *d* = 0.14 in the auditory modality and significant, albeit with low effect size, *t*(47) = 2.305, *p* = 0.026, *d* = 0.33 in the visual modality. For non-linguistic material, the difference from zero was significant and important, with a moderate effect size, both for the auditory, *t*(47) = 3.57, *p* < 0.001, *d* = 0.52, and for the visual, *t*(47) = 2.944, *p* = 0.005, *d* = 0.42, modalities. The result pattern is displayed in Figure 4 (adapted from Ordin et al., 2021).

Whether lenient criterion fully accounts for the difference in confidence ratings on foils and constituents remains an open question because the bias to endorse the test tokens (i.e., to respond “yes”) is not different between modalities and material types (Ordin et al., 2021), and that significant deviations from zero were not observed in the auditory condition on linguistic material, yet the confidence pattern conditioned to the stimuli type (*foil* vs. *actual constituent*) was the same across all modalities and material types. Besides, the difference in confidence ratings assigned to correct and wrong responses on trials, in which constituents were presented is larger than the difference in confidence on correct and wrong responses on trials, in which foils were presented: Δ_foils < Δ_constituents, *p* < 0.001 in paired *t*-tests for each modality on both linguistic and non-linguistic material.

Taken together, the data suggest that metacognitive monitoring is differentially affected on constituents and foils, with metacognitive monitoring on trials when constituents are presented being stronger than on trials when foils are presented (weak version of the hypothesis), or metacognitive failure when foils are presented (strong version of the hypothesis, which required further empirical testing).

## Discussion

We found that the confidence ratings are discriminative of correct and incorrect responses in the expected manner (i.e., higher on correct than on incorrect responses) only on trials when people had to recognize words or recurrent sequences from the familiarization input. On trials in which foils were presented the confidence ratings revealed the reverse pattern. This was confirmed across four experimental sessions: in visual and in auditory modalities both on linguistic and non-linguistic material. This suggests that metacognition is efficient in those cases when people need to evaluate how well they have learnt something. When people need to report how sure they are in what they have not been learning, metacognition fails (or we fail to capture metacognitive efficiency based on retrospective confidence ratings). This should be considered in experimental design, in terms of wording for the tasks and structure of the trials.

Intriguingly, there is no observable difference in confidence assigned to foils of different types, although random foils should be easier to reject because people need to detect the novel element at the triplet-initial position in order to be able to reject the foil, the confidence in decision should increase once the second element, also violating the expectations, is processed, leading to higher confidence on rejected foils. On ordered foils, besides positional information, relational information (which element is expected given preceding one(s). This can be calculated once the first element has happened on a test token, giving less time for confidence accumulation toward the end of the triplet. According to the Relational Complexity Theory (Halford et al., 1998), in the process of conceptual segmentation (i.e., during the learning stage of the artificial language learning experiments), new representations of segmented units are formed by reducing complexity *via* collapsing dimensions (sources of variation). A new holistic representation is easier to process, but different sources of variation within the segmented and consolidated unit can no longer be unpacked. Hence we did not observe the effect of difference in complexity on confidence. However, to further explore the potential relation between complexity and confidence, in the future studies we will need to focus on the foils that violate relational information (ABC–target vs. ABD–foil), introducing multiple dimensions (sources of variability) of complexity.

Why we observed a reverse result pattern in how confidence is assigned on trials with foils remains unclear. We expected the confidence being not discriminative between correct and incorrect responses, which would indicate a poor metacognitive efficiency. Neither did we expect any difference in the number of correct responses per confidence level on the trails with foils. However, on foils trials participants consistently assigned significantly higher rating to wrong responses. We propose several explanations why our expectations were violated on foils trials.

(1) Correct response on foils is rejection, while correct response on words is acceptance. Rejection and acceptance might rely on differential neuro-cognitive mechanisms, and monitoring of these mechanisms might also differ, leading to differential result patterns on trials with foils and words.
(2) We have twice as many foils as words in each session; hence people should reject items twice as frequently as accept items. However, given the dual choice, participant might have expected an equal distribution of trials when they need to accept and reject presented test tokens. Thus, with each new token that had to be correctly rejected, participants’ confidence in their response might decrease.
(3) Accepting items elicits higher confidence overall, leading to higher confidence on correct responses on trials with words and on incorrect responses on trials with foils. In other words, we can evaluate the changes in mental states based on evidence, but not absence of evidence. This is an important confounding factor that also undermines the experimental design that incorporates the analysis of “yes” versus “no” responses with the analysis of responses on items (or rules for constructing novel items) that have been learnt and those that have not been learnt. A potentially promising approach might be based on the differences in searching or decision-making time on the trials in which the uncertainty that the “award” is expected is high, versus trials in which the uncertainty is low).

Another important consideration is the degree of conscious awareness into metacognitive judgments. Metacognition relies both on conscious and unconscious processing (Nelson, 1996; Kentridge and Heywood, 2000; Jachs et al., 2015). The nature of this task diminishes the contribution of the latter because people, when explicitly asked to rate their confidence, are more likely to consciously contemplate on their decisions (Ordin and Polyanskaya, 2021). This might highlight awareness of what is learnt and known (Drigas et al., 2023), but hinders awareness of what has not been learnt, yielding different result patterns in terms of retrospective confidence on trials with foils and words. Alternative procedures are also necessary to study the contribution of unconscious processing into metacognitive efficiency because the ability to discriminate on the basis of confidence is often assumed to rely on conscious awareness of stimuli (Smith et al., 2003; Persaud et al., 2007). Explicit instructions to evaluate one’s performance with confidence ratings skew the balance between conscious and unconscious processes in metacognition in favor of the former.

These methodological considerations do not undermine the usefulness of the signal detection theoretic approach to modeling metacognition using confidence ratings. Hits attract higher confidence rating than false alarms, but correct rejections attract lower confidence rating than misses. However, the difference in confidence between hits and false alarms is greater than between correct rejections and misses, thus the modeling approach nevertheless provides useful information is we need to compare metacognition between groups, between tasks, or between modalities/domains. The signal detection modeling approach offers a clear advantage when comparing metacognition across tasks, domains, and modalities that vary in terms of task performance and metacognitive bias, which affect alternative measures of metacognition based on retrospective confidence (Masson and Rotello, 2009; Maniscalco and Lau, 2012; Barrett et al., 2013). Also, it provides a clearer link to conscious awareness because M-ratio effectively shows the extent to which a metacognitively ideal observer is aware of his task performance. Also, as meta-D’ and D’ are measured in the same units, sensitivity in the task and metacognitive sensitivity can be explicitly compared, and given larger difference in confidence between hits and false alarms than between misses and correct rejections, the SDT will nevertheless yield valid results. However, care should be taken in regard how questions are asked and whether people are indeed asked to evaluate what they know rather than what they do not know (in the latter case, differences between conditions might be diminished due to reverse confidence patterns on hits and false alarms versus misses and correct rejections. In statistical learning experiments, it might be useful, for example, to implement alternative forced-choice methods, when people need to select between a foil and a word, which of the two tokens is embedded into familiarization stream (e.g., Ordin et al., 2020; Ordin and Polyanskaya, 2021). Such trials always include evaluation of what people (supposedly) know. Avoiding mixing trials that require estimating positive evidence and trails that require estimating absence of evidence will increase the strength of the SDT analytic approach.

## Data availability statement

Publicly available datasets were analyzed in this study. This data can be found here: https://doi.org/10.6084/m9.figshare.20995345.

## Ethics statement

A secondary dataset that was originally collected for other research purposes was used for this study. Ethical review and approval, and written informed consent, were not required for re-analysis of the existing dataset in accordance with the national legislation and the institutional requirements.

## Author contributions

The author confirms being the sole contributor of this work and has approved it for publication.
